# Mining and Environmental Health Disparities in Native American Communities

**DOI:** 10.1007/s40572-017-0140-5

**Published:** 2017-04-26

**Authors:** Johnnye Lewis, Joseph Hoover, Debra MacKenzie

**Affiliations:** Community Environmental Health Program, College of Pharmacy, University of New Mexico Health Sciences Center, 1000 Stanford Drive NE, MSC095360, Albuquerque, NM 87131-0001 USA

**Keywords:** Abandoned mines, Native Americans, Environmental health, Environmental justice, Heavy metals, Environmental policy

## Abstract

**Purpose of Review:**

More than a century of hard rock mining has left a legacy of >160,000 abandoned mines in the Western USA that are home to the majority of Native American lands. This article describes how abrogation of treaty rights, ineffective policies, lack of infrastructure, and a lack of research in Native communities converge to create chronic exposure, ill-defined risks, and tribal health concerns.

**Recent Findings:**

Recent results show that Native Americans living near abandoned uranium mines have an increased likelihood for kidney disease and hypertension, and an increased likelihood of developing multiple chronic diseases linked to their proximity to the mine waste and activities bringing them in contact with the waste. Biomonitoring confirms higher than expected exposure to uranium and associated metals in the waste in adults, neonates, and children in these communities.

**Summary:**

These sites will not be cleaned up for many generations making it critical to understand and prioritize exposure-toxicity relationships in Native populations to appropriately allocate limited resources to protect health. Recent initiatives, in partnership with Native communities, recognize these needs and support development of tribal research capacity to ensure that research respectful of tribal culture and policies can address concerns in the future. In addition, recognition of the risks posed by these abandoned sites should inform policy change to protect community health in the future.

## Introduction

Contamination of soil and water by waste from more than 160,000 abandoned hard rock mines throughout the Western USA (operationally defined to include Arizona, California, Colorado, Idaho, Montana, Nevada, New Mexico, Oregon, South Dakota, Utah, Washington, and Wyoming) has created a legacy of chronic exposures to metal mixtures in Native American communities. Biomonitoring in the Strong Heart Study cohort which included Native Americans from these Western states supports increased metal exposures by identifying unique patterns of elevated metal mixtures relative to several other races in urban, suburban, and rural settings [1]. The political and social context in which these exposures developed highlights a history of environmental injustices based in clashes of cultural values, and political and ethical failures to support negotiated treaty rights. Understanding the health impacts of these exposures is complicated by a lack of understanding of the toxicity of metals in complex mixtures in any population, exacerbated by a general lack of environmental health studies in Native populations in particular. In addition, many of the Native communities in proximity to these waste sites have numerous risk factors associated with disparities in health outcomes such as poverty, educational status, infrastructure, and frequently, compromised underlying health status. Further complications to predicting toxicological responses arise from the traditional and subsistence lifestyles of many Native communities that create distinct exposure patterns not captured in the assumptions of standard suburban, recreational, or occupational exposure scenarios used for risk assessments [2]. Traditional lifestyles, such as eating or harvesting local plants for sustenance, ceremonial or medicinal purposes, or drinking from historically used water sources may result in greater than predicted exposures of Native Americans to mine wastes now contaminating these sources.

The impacts of the waste on tribal nations are not likely to resolve for generations to come. In addition to the number of sites and estimated clean-up costs that exceed any available resources, the sheer volume of wastes at these sites is a barrier. Laguna Pueblo’s Jackpile Mine Superfund site alone is greater than 34 km^2^ including disturbance of more than 12 km^2^ [3]. Geochemical and physical factors complicate understanding mobility and challenge technological remediation strategies as the complexities in mixture composition and geochemistry produce varying results across sites. Removal of waste is complicated by the hazards inherent in transport of such large volumes of hazardous materials through communities, and in the case of uranium, by the fact that the mixture of toxic and radioactive metals creates a mixed waste with limited disposal options. This paper will highlight the framework in which these health, political, social, and environmental factors converge to create ongoing uncertainty about tribal health and to discuss new initiatives to reduce these uncertainties for generations likely to face these risks well into the future.

## Treaty Rights vs. Mining Interests on Tribal Lands

Through various treaties developed between Native American tribes and the US Government, the tribes ceded vast amounts of their traditional land base in exchange for recognition of their sovereign status to self-government and commitments to ensure protection of their health and culture. Through these treaties, certain regions of the country were set aside as Tribal “Reservations”—lands over which a tribe had sovereignty. As mineral resources were identified on those lands, ethical and legal responsibilities set in the treaties were abrogated by the US government to provide access for mining by successively shrinking the area of lands designated to each tribe; by legislating in the 66th Congress the leasing of tribal lands in the Western USA by individuals and corporations for purposes of mineral extraction for 20 years, renewable in successive 10 year periods; and by prohibiting in a 1919 law the creation of any future Reservations on Public Lands through executive orders of the President [4]. In *If You Poison Us*, Peter Eichstaedt’s comprehensive review of congressional debates and policies leading up to this legislation describes discussions indicating that the creation of Reservations by Executive Order on lands holding such vast reserves of minerals would never have been allowed had Congress been aware of those reserves [5••]. In 1902, Indian Affairs Commissioner William Jones argued that land should not be set aside exclusively for Native American use and the more than 200,000 km^2^ currently in Reservations should be “thrown open as rapidly as possible”, acknowledging that the treaties made it necessary for Congress to “treat with them” before opening their lands to settlement, and therefore Congress should use some arbitrary means to open the land [6•].

The 1919 legislation provided for 5% of the net value of the extracted resources paid to the USA “for benefit of the Indians” to be deposited in the Treasury to the credit of those Indians having tribal rights to lease land, but at all times subject to “appropriation by Congress for their benefit”. However, documented shadow accounting by mining companies reduced that net value and therefore the amount paid into those accounts [5••]. The continued disregard for the ethical and legal guarantees of the treaties resulted in long-term mismanagement of Native American Tribal Trust funds by the US government and further limited tribal benefits. Nearly a century later, between 2009 and 2016, the US Attorney General and Secretary of the Interior reached settlements with more than 100 tribes totaling $3.3 billion USD for mismanagement of monetary and natural resource assets held in trust by the USA for the benefit of the tribes [7]. These settlements, however, do not come close to addressing the mining legacy. A recent $1 billion USD settlement from Tronox awarded to the Navajo Nation has been estimated sufficient to address remedial action of only ∼10% of the 3.6 billion kg of uranium mine waste on Navajo Nation alone [8]. The 520 abandoned uranium mines on Navajo represent less than 12% of the more than 4000 abandoned uranium mines in the Western USA, and an even smaller fraction of the more than 161,000 abandoned hard rock mines in the Western USA. These numbers provide a perspective on the scope of the problem being faced by tribal communities.

## Scope of the Problem and Ongoing Threats

**Fig. 1:**
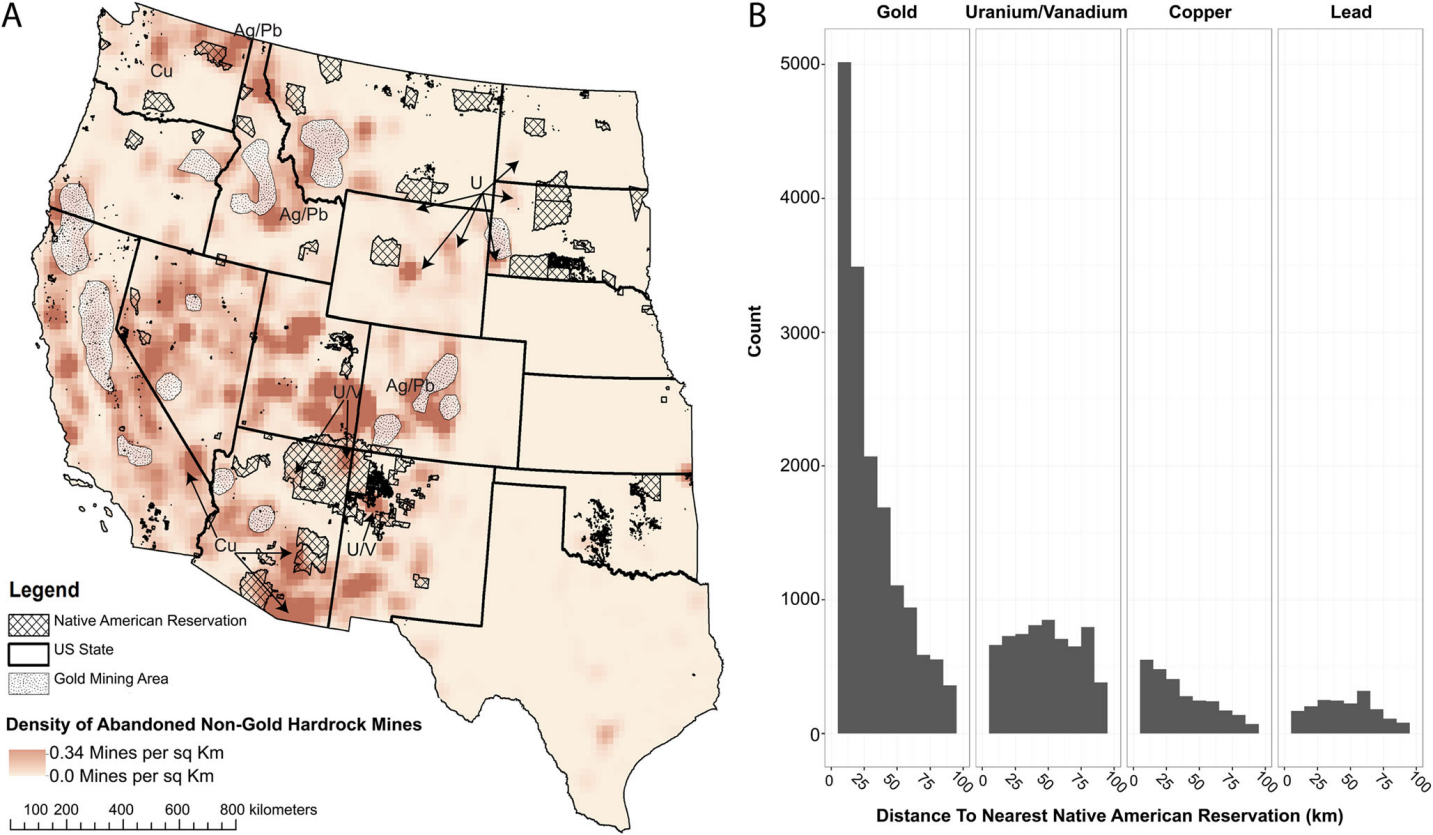
**a** Density of hard rock metallic mines in the Western USA. Native American Reservation land is indicated by *hatched polygon areas*, and mine densities are associated with intensity of *red hues*. The predominate commodity type is also indicated on the map by its *chemical symbol* (*Au* [Gold]; *Pb* [Lead]; *U* [Uranium]; *V* [Vanadium]; *Cu* [Copper]). **b** Histogram of distance between hard rock mines (by primary commodity type) and the nearest Native American Reservation

As a result of the disregard of treaty rights, lands on and proximal to Native lands in the Western states (Fig. 1) were extensively mined for metals such as gold, silver, lead, copper, molybdenum, and vanadium since the mid-1800s and uranium since the 1940s. As deposits played out and demand for specific metals declined, the mines were abandoned—resulting in more than 160,000 abandoned hard rock mines (not including coal) in the Western states [9], more than 4000 of which are abandoned uranium mines (AUMs) [10]. Today, more than 4.1 million Native Americans live in the Western USA, 478,000 on Reservation lands [11]. Combining US Census information [11] and a gridded population dataset [12], we estimated that more than 600,000 Native Americans live within 10 km of an abandoned mine (Table 1). Potential associated contaminants of concern for Native Americans within 10 km based on the primary metal mined are listed in Table 1. In addition, depending on the process, chemicals such as cyanide, sulfate, nitrate, and others used in ore extraction could remain in the waste or water sources. Population estimates based on census data assume inclusion of tribal members in the Census pool. While estimates of accuracy of count for the most recent census are not yet available, previous estimates have indicated an undercount of Native Americans ranging from 5% [13] to nearly 20% [14].

**Table 1:** Summary of common metals and metalloids associated with waste from mines and an estimate of the number of Native Americans living within 10 km of each mine type

Hard rock metal mine type	Common metal contaminants of concern	Estimated count of Native Americans living <10 km from mine type
Gold	Arsenic, cadmium, cobalt, copper, mercury, nickel, lead, zinc [15, 16]	417,846 people
Uranium/vanadium	Arsenic, copper, molybdenum, nickel, selenium, uranium, vanadium [17, 18]	286,346 people
Copper	Arsenic, cadmium, copper, iron, nickel [19–21]	243,722 people
Lead	Arsenic, cadmium, chromium, manganese, lead, zinc [22–25]	116,925 people

Additional associated exposure sources include ore transfer stations, waste piles, mill tailings piles, and areas affected by spills, resulting in more than 500,000 discrete contamination sources [9]. Sites are most often unmarked, unfenced, and located only through historical memory or mining records. Using available geospatial information on mineral extraction from the US Mineral Resources Data System [26], Fig. 1 visually highlights the proximity of reservation land to these mining regions.

Although tribal control of mineral resource leasing and extraction has strengthened during the twentieth century due to laws such as the Indian Mineral Development Act of 1982 and the 2001 Bureau of Land Management Rule 3809 [27], the General Mining Law of 1872 remains in effect as the overarching regulation. The location of abandoned mines within tribal watersheds and the lack of environmental protections in the 1872 Mining Law has left a legacy for exposure not only through direct contamination of tribal lands, but through continuing drainage of contaminated mine water. Impoundments of untreated mine waste and water are designated as waste treatment systems, which exempts many bodies of water from Clean Water Act regulations [28]. This designation supported by the 1872 Mining Law allows discharge of untreated mine waste into surface water and has resulted in contamination of an estimated 40% of the headwaters of Western US watersheds [29], water sources still relied on by virtually all Native American tribes for survival and cultural preservation [28].

The Gold King Mine Spill in 2015 was but an example of the implications this legacy poses throughout watersheds in the West. In that spill, 11,300 m^3^ of contaminated mine water breached containment from the abandoned Gold King Mine in Southern Colorado, carrying lead, arsenic, and other metals (see Table 1) through Ute and Navajo tribal lands as well as non-tribal lands along the San Juan River [16], leading Navajo Nation to file a lawsuit against USEPA [30]. Releases from abandoned gold mines have also contaminated waters and lands of tribes including, among others, Fort Belknap in north-central Montana and numerous tribal lands in California, Colorado, Nevada [31–33] and South Dakota (see “Case Study 1” below).

The threats to tribal lands, however, are not only from legacy mines. New mines continue to be fought by tribal communities as illustrated by legal actions taken by the Western Shoshone Tribe in Nevada [31], the Menominee Tribe along the Michigan-Wisconsin border [34] and Navajo communities [35]. Pressure to open new uranium mines led Navajo Nation to pass the Natural Resources Protection Act of 2005 banning uranium mining and processing within Navajo Indian Country as the Fundamental Laws of the Diné indicate substances in the Earth harmful to the people should not be disturbed [36].

## Sociodemographic and Infrastructure Risk Factors in Native Communities

Without proper understanding of toxicity and factors controlling it in Native American populations, the risks represented by these wastes remain ill-defined. Native Americans have lower life expectancy and more health challenges than other populations in the USA. Data compiled by Indian Health Services (2007–2009) and other entities illustrate the significant health disparities observed within these communities (Table 2). These disparities are frequently attributed to genetic susceptibility, and while some studies show mutations in individuals with a specific disease, the lack of population genetic data defining prevalence questions this assumption [37••]. The persistence of disparities in virtually all prevailing diseases across multiple language groups suggests other contributors to these outcomes, such as social determinants, underexplored disparities in environmental exposures and response to toxic insults, or gene-environment interactions [38].

**Table 2:** Summary of key health disparity measures for the Native American population in the USA

Health measure	Native American disparity
Suicide	Rate is 2.5 times higher for Native youth [39].
Infectious disease mortality	40–60% higher than all races in the USA [40]
Diabetes and liver disease mortality	2.8–4.7 times higher than all races in the USA [41]
Infant mortality	28% higher than non-Hispanic whites [42]
Overall mortality (0–44 years old)	2.23–2.69 times greater than non-Hispanic whites [43]
Birth defects	50% higher prevalence in Native Americans [40]
Life expectancy	4.4 years lower for Native Americans [39, 40]

Experimental and epidemiologic data support the association between exposure to toxic metals and increased risk for a variety of negative health outcomes including kidney and cardiovascular disease, neurocognitive disorders, and various cancers in both Native and non-Native populations [44–56]. Cancer mortality rates for the Native American population increased over a 20-year period (1990–2009), while decreasing for Whites during the same time [43]. Two prospective studies have specifically linked exposure to the metals cadmium and arsenic to cancer mortality in Native populations [53, 56]. Preliminary epidemiologic data document nearly 4 out of every 10 residents of the Wind River Reservation of Eastern Shoshone and Northern Arapaho have a blood relative who has died of cancer [57]. Wind River was the site of a former uranium mill and the remediated waste pile remains in the community. The preliminary report falls short of making a direct association but reinforces their concern that the waste is adversely affecting health [57, 58]. In 1988, US DOE found soil, surface water, and shallow groundwater on Wind River Reservation were contaminated with uranium, radium, and thorium and chose to use natural attenuation to reduce the contamination over time. However, following floods in 2010, monitoring wells showed uranium spikes as high as 100 times the USEPA Maximum Contaminant Level (MCL) for drinking water (0.03 mg/L) [58]. Despite this and other reports of contamination linked to increased cancer incidence and other negative health outcomes, there remains a paucity of population studies in these Native communities. Moreover, our understanding of exposure-toxicity relationships in Native communities continues to be limited by our general lack of understanding of the toxicity of any complex mixtures. Even determination of dose in Native communities can be grossly underestimated by neglecting traditional cultural practices resulting in frequent and direct contact with contaminated lands and waters, and a greater, sometimes 100%, reliance on traditional and locally grown foods [2].

Disparities in tribal infrastructure further exacerbate risk. Nearly 14% of Native households lack access to a public water system compared to 0.6% of the USA as a whole; with some tribes lacking access for more than 30% of their populations [59], creating a greater reliance on unregulated sources. Uranium MCLs are exceeded in more than 13% of unregulated wells on Navajo Nation, with arsenic exceeding the MCL in 15% and co-occurrence in 7% of wells [60]. Concentrations are strongly associated with distance from abandoned mines. Moreover, when regulated public water is available, drinking water systems in Indian country experience “significant violations” or health-based violations of  Safe Drinking Water Act regulations twice as frequently as other systems [61], exceeding MCLs for various metals, polycyclic aromatic hydrocarbons, trihalomethanes, pesticides, and nitrates. Two months prior to detection of lead in the Flint, MI, water supply, a *regulated* community drinking water supply serving the Navajo community of Sanders was found to have exceeded uranium MCLs for more than a decade as a result of migration of contaminants from a 1979 uranium mill tailings spill [62]. Although state officials were aware of the problem, in contrast to Flint, no notification to the community occurred, and no federal outcry or resources resulted. These challenges and others demonstrate the need for infrastructure [63] and that the gap in fundamental infrastructure sets a background exposure upon which other environmental exposures and health disparities are overlaid.

## Case Study 1: Black Hills Gold and the Sioux Nation

For the nine tribes of the Great Sioux Nation, the Black Hills are sacred lands of their traditional homeland in the northern Great Plains. Following the Battle of Little Big Horn, the 1868 Fort Laramie Treaty between the US government and Sioux Nation guaranteed them “undisturbed use and occupation” of the Black Hills and stated that no treaty for the cession of any part of the reservation would be valid unless signed by three-fourths of the adult male Sioux population. However, when gold was discovered, those treaty rights were abandoned by US confiscation of the Black Hills in the 1877 Act, signed by fewer than 10% of the adult male Sioux population, to allow for mining. Over the next century, mining companies extracted billions of dollars in metals from these lands. Production from 1875 to 1971 reported more than 1.1 million kg of gold extracted, which even at $1 USD per gram would exceed $1 billion USD. The Sioux never accepted the legality of the Black Hills confiscation and sued the US government for return of the land. In 1980, the US Supreme Court upheld a decision to support the Sioux claim that US government’s decision to occupy the Black Hills constituted a “taking of tribal property” with an implied obligation for the government to make just compensation. Rather than returning the land, however, the US Supreme Court awarded the nine tribes of the Great Sioux Nation $102 million USD as compensation for their loss. The Sioux have never collected this payment, which remains in trust and now exceeds $1.3 billion USD [64].

Why would some of the poorest tribes in the country, with unemployment rates as high as 80%, turn down such a large sum of money? The award of financial compensation misses many key points central to the disputes between the tribes and the federal government. First, the suit was never about money but about the return of lands central to the spirit of the Sioux Nation. Second, the value is small relative to the profit made from both the land and resource extraction in the intervening years. Third, to take money for the land is acknowledging a real estate transaction, and in the words of former Oglala president Teresa Two Bulls “If we accept the money, then we have no more of the treaty obligations that the federal government has with us for taking our land, for taking our gold, all our resources out of the Black Hills … we’re poor now, we’ll be poorer then when that happens,” [64]. While the Sioux and the federal government continue to seek a compromise, the legacy of the Black Hills mining continues to pose concerns for the health of the Sioux.

As illustrated in Fig. 1, more than 10,000 abandoned gold mines are on or near Native American Reservation lands throughout the Western USA. In the Black Hills, there are hundreds of gold mines but few as profitable as the Homestake Mine near Lead, which produced more than 964,000 kg of the 1.1 million kg reported for the Black Hills from 1875 to 1971 [65]. Homestake discharged high concentrations of arsenic from their mill directly into the Cheyenne River drainage for decades [66] contaminating 29 km of downstream creeks that received Superfund action for arsenic contaminated soil, sediments, and water. Although the site was removed from the National Priorities List in 1996, there remains a band of arsenic-containing sediment in the Cheyenne River banks and floodplains that form the southern boundary of the Cheyenne River Sioux Tribe Reservation (more than 250 km downstream) [67]. While the arsenic-contaminated sediment in the banks has been buried by years of deposition since the closure of the mine in 2002, intermittent floods during operational years deposited contaminated sediment that remains in surface soils of lands used for agriculture, ranching, and ceremonial purposes as far as 1 km from the river (*personal communication*, Dec. 2016, C. Ducheneaux, Department of Environment and Natural Resources, Cheyenne River Sioux Tribe).

Within the Cheyenne River Sioux, antinuclear antibody (ANA) prevalence exceeds that found in the US population, with occurrence predicted by the proximity to the arsenic deposits [68]. While the presence of ANA does not alone indicate frank autoimmune disease, an associated elevated prevalence of several specific autoantibodies was also observed [68]. ANAs serve as early serologic markers in a variety of autoimmune diseases [69–71] and have also been linked to mercury exposure from Amazonian gold mines, to consumption of fish contaminated with methylmercury, and to drinking arsenic contaminated water [72–76].

The arsenic is located in the floodplains where gathering of fruit for sustenance and herbs for medicinal, traditional, and ceremonial practices has been documented as well as other traditional land use practices that create direct exposure pathways through ingestion, inhalation, and dermal contact. These uses raise ethical and policy issues around how exposure and health are evaluated. If standard exposure assessment assumptions interpreted through extant health research are applied to a Native community practicing a traditional lifestyle, such as the Cheyenne River Sioux, results may underestimate exposure [2]. Secondly, not only are data on response to toxicants limited for Native Americans, the definition of health itself may differ substantially between the cultures with regulatory agencies interpreting health from a Western disease model and the tribal population using a more holistic view based their belief of interconnection between people, their ecosystem, and their well-being [77••]. Our team is currently working with the CRST to understand the mobility of the Homestake arsenic; evaluate human exposure through ceremonial, traditional, ranching, and recreation activities; and assess the contribution of exposure to the high prevalence of autoimmune markers observed in those living in the floodplain [68].

## Case Study 2: Uranium and the Navajo Nation

Uranium mines present a special subset of abandoned hard rock mines resulting from the Cold War and associated political stressors. Although reservations encompass only 5.6% of land area in the Western USA, approximately one in five uranium mines are located within 10 km of an Native American Reservation with more than 75% (more than 3200 of the 4600) within 80 km (Fig. 1). This does not include lands traditionally used by the tribes or lands feeding tribal water- and airsheds. Figure 1 highlights areas where uranium was the dominant ore extracted and illustrates that multiple tribes are affected by AUMs, including Navajo Nation in the Four Corners region, multiple Sioux tribes in the Black Hills region, Northern Arapahoe and Eastern Shoshone at Wind River in Wyoming, and several tribes including the Spokane in Washington State.

The development of the atomic bomb and the subsequent nuclear arsenal that fueled the Cold War led to prioritization of uranium mining above protection of human health, as well documented by Eichstaedt in *If You Poison Us* [5••]. Vanadium, which occurs in combination with uranium in carnotite, was mined on Navajo as early as 1902 to support steel hardening. The Manhattan Project’s need for uranium made the repurposing of these mines expedient and led to government action to promote uranium exploration without regard for protection of human health. The hazards of radiation were recognized since the 1920s, and increased respiratory disease and lung cancer in European uranium miners was well known by the US Atomic Energy Commission (AEC) and others from the findings of lung cancer and respiratory disease in European miners. However, the AEC was reluctant to set standards within mines or take any other measures to protect miners’ health, fearing that public awareness of the dangers in the mines would limit their ability to obtain uranium. Following the US Public Health Service’s (PHS) requests to set safety standards, the AEC set standards of 10 pCi/L in all operations *except* uranium mines; after lengthy debate, a standard of 100 pCi/L in uranium mines was set in 1967—23 years after the beginning of uranium mining on the Navajo Nation and 17 years after the first US Public Health Service uranium miners’ health study identified respiratory risks. It would be another 33 years before these details were made public and the US Congress passed the Radiation Exposure Compensation Act (RECA) that would begin a process of compensating those affected. Compensation of Navajo miners was slowed significantly, however, as a result of barriers in the law requiring documentation of births, marriages, and death records not routinely kept within the tribe and the restriction of compensable health effects to lung cancer only. The law has been revised only once, in 2000, to incorporate additional compensable diseases and include uranium mill workers [78], while additional revisions to compensate miners who worked after the government turned over mine safety to the private sector in 1971 remain in limbo.

As late as 1991–2005, 25% of the deaths in the 4137 former uranium miners followed by US PHS were attributed to lung cancer, with Native miners at triple the expected rate [79]. Native miners bore a disproportionate burden of non-malignant respiratory disease as well. Their rate of pneumoconiosis was as much as fourfold higher than in non-Hispanic whites (NHW), and they also had an increased risk of restrictive lung disease and decreased FEV_1_ [80]. For Navajo miners, underground uranium mining was the primary contributor to non-malignant disease including obstructive lung disease, while in non-Native populations, smoking was the major contributor. More than 70% of Navajo miners had never smoked (compared to 23% of NHW miners) [80]. However, compensation through the government’s Radiation Exposure Compensation Act, discriminated against Native miners by requiring, in addition to radiographic evidence of lung damage, a second measure of spirometry less than 75% of a comparison value derived from a NHW population. Clinical researchers demonstrated that using that criterion to determine loss of function resulted in exclusion of 24% of Native miners who would qualify with a 75% reduction in lung function if Native American-based function formulas were used [80]. Other studies concluded that Colorado Plateau Native American uranium miners disproportionately suffer excess mortality risks from lung cancer [81, 82]. Native Americans were a substantial portion of the underground workforce, and it has been argued that the observed differences in effect related to a higher exposure. Disease rates reported above, however, were corrected for exposure, and Native miners were shown to have significantly *lower* average lifetime mining exposure than NHWs and Hispanics [80]. As many as 4000 Navajo men worked in the uranium mines and mills and hundreds of Navajo miners died and continue to die of lung cancer and other respiratory disease [83, 84], essentially wiping out a generation of Navajo men in some communities and leaving a legacy of psychological and environmental trauma [84]. The increased toxicity to Native miners underscores the potential for unique sensitivities to toxicants within the Native community as compared to all races results, questioning the derivation of standards on the basis of data collected from other populations.

While the fights over RECA proceeded, people living in Navajo communities continued to be chronically exposed to waste from the 520 abandoned uranium mines, 4 abandoned uranium mills, and more than 1100 waste sites [85] in and proximal to their communities. Uranium mine waste presents a unique hazard resulting from the associated radioactive decay chain introducing additional contaminants including radium, thorium, and radon to the already complex mixture of associated metals often including, for example, arsenic, cadmium, and lead [86]. The lack of regulation and security of waste associated with these facilities and abandoned sites, as well as lack of concern during the operational phase led to the use of contaminated material in the construction of homes [85], as well as contamination of water sources potentially used for drinking [59]. Looking at >1300 Navajo community members with and without exposure to uranium mine waste, we find a significant strong association between exposures occurring during the active mining era (1940s–1980s) and later development of kidney disease, exposure more than doubling the odds ratio. For those exposed to legacy waste, controlling for the mining era exposures, exposure is significantly associated with an increased likelihood of hypertension and of developing one or more chronic diseases including hypertension, diabetes, and kidney disease [87••]. Serum from a subset of >200 individuals from that cohort, representing a full range of exposure, showed a significant inverse relationship between inflammatory potential in cellular bioassays and distance from legacy waste sites consistent with induction of pathways that contribute to atherosclerosis and cardiovascular disease [88]. While risk factors traditionally considered for these diseases clinically were also significant predictors, these results underscore the need for clinicians to probe for information about exposure as well in identifying those at risk.

## Ongoing Concern for Future Generations

Among Native communities, there is concern about not only the health of people living in impacted communities today but also the health of generations yet to come. The Women of All Red Nations (WARN) worry that uranium mining may contribute to high rates of miscarriage and reproductive cancers observed among Lakota women [89]. While studies of direct impacts of mine waste exposures on tribal lands have been extremely limited, several recent initiatives are beginning to fill these gaps.

A 1981 study in Navajo babies showed that congenital anomalies, developmental disorders, and other adverse birth outcomes were associated with maternal proximity to uranium mining operations, tailings, or mine dumps [90]. Uncertainties in these results were not followed up until 2010 when the Navajo Birth Cohort Study (NBCS) [37••, 91] was initiated as part of a Congressional Five-Year Plan to Address Uranium Contamination on Navajo [92]. The NBCS is a prospective assessment of the effect of exposure to uranium and co-occurring metals in mine waste on birth outcomes and neurodevelopment through 1 year of age conducted as a partnership with the University of New Mexico, Southwest Research Information Center, Navajo Nation Indian Health Service, Navajo Nation Department of Health, and funders CDC/ATSDR. This is the first prospective birth cohort addressing the impacts of environmental contamination on birth outcomes in a Native population. Preliminary data confirm exposures to uranium prenatally and over the first year of life. The recently funded NIH National Environmental Influences on Child Health Outcomes (ECHO) initiative will include both the NBCS and a Sioux cohort in this national synthetic cohort of more than 50,000 children, allowing assessment of the relationship of metal exposure, neurodevelopment, and other outcomes through at least age 5. ECHO will also allow determination of unique sensitivities within these tribal populations through comparisons of exposure-toxicity relationships across cultures, races, and lifestyles.

Two of five NIEHS/NIMHD/USEPA P50 Centers of Excellence in Environmental Health Disparities funded in 2015 focus on multiple Native American populations and metals exposures. These centers will also help to identify unique exposure pathways, understand the biogeochemical characteristics of metal mixtures, and document mechanisms of toxicity.

These multigenerational studies using common methods and involving Native American communities in data collection and interpretation at all stages begin to answer the longstanding questions on health, and, importantly, to build capacity for investigation of these questions by tribal researchers.

## Ethical, Scientific, and Policy Challenges to Increasing Native American Research

To fully understand the impacts of abandoned mine waste and to inform policies that protect tribal populations from future adverse health impacts, the body of scientific studies will need to incorporate more tribal studies, incorporate cultural and traditional practices into assessments of toxicity, and better understand unique responses in these populations. This broader inclusion, however, comes with its own challenges.

The recognition of treaties granting sovereign status to tribal nations within the USA brings with it rights of tribes to control the research process through their own institutional review boards, to mandate that all data and specimens generated through research remain the property of the tribe, and to mandate conditions for data sharing and certain types of analyses, such as genetic analyses. Recent requirements of the US National Institutes of Health (NIH), the major funders of health research in the USA, require sharing of all data collected with public funds, and often require investigators to obtain consent for genetic analyses, creating cultural conflicts between the tribes and research funders. Models are emerging, however, that show these issues are not insurmountable. Alaska Natives have developed a biospecimen and data repository that stores all samples and data collected on Alaska Natives and controls future use through a board on which Alaska Natives are in the majority [93]. NIH recognition of the importance of these concerns is supported by their creation of a Tribal Research Office within the office of the director, currently led by a Native molecular biologist [94].

Tribal demands previously seen as challenging, such as involvement of tribal communities in all phases and aspects of research; insistence on honest communication of research goals and progress throughout the study; and ensuring research has benefits for tribal communities by informing policy for improved health care and prevention of future health impacts are now consistent with best practices for environmental health studies as discussed in the Ethics Guidelines for Environmental Epidemiologists [95]. While recognition of the need to include tribal populations in research has grown in the federal agencies and research community, grant reviewers even recently have expressed ethical concerns over funding research in Native American populations because it will not be generalizable. Additional education and guidance to point out the ethical fallacy in the current practice of generalizing dominant culture results to policies affecting tribal populations may be necessary before equity is achieved.

## Conclusions and Future Research Directions

The abandoned mines on and near Native lands in the Western USA are but one in an ongoing series of insults. The sheer number of mines and the volumes of waste guarantee that the legacy will persist for generations to come. In addition, the mines present only one chapter in the history of environmental injustices on Native lands. Similar articles could be written focusing on impacts to tribal lands from coal strip mining, from the legacy of military bases, and from oil and gas development. The support from thousands representing tribes across the country for the Standing Rock Sioux camp opposing the Dakota Access Pipeline in 2016 is indicative of the common theme felt by tribes not only in the Western USA but throughout the country and the world where treaty rights to protect health and culture have been continually challenged and eroded in favor of resource development.

In spite of the recognized threats to tribal health posed by these ongoing toxicant exposures, and the demonstrated risk factors that put tribal populations in a position of increased vulnerability, research to understand the health impacts has been hampered by the small sizes of the communities, reviewers concerns about generalizability of results, and in some cases by the distrust of research within tribal communities who have witnessed researchers build careers but leave nothing behind to improve the health of the population. In contrast to the identified risks, the strength and support derived from the maintenance of culture, ceremonies, and traditional lifestyles may also provide a resiliency to offset some of the adverse effects. Understanding how these factors interact is critical to designing effective interventions to protect health benefitting all populations.

The history of mining in the Western USA and the legacy that affects so many Native American communities highlight the need for not only better inclusion of tribal populations in toxicologic studies but also in using these data in the development of standards that ensure the cleanup of the sites is truly protective of the health of the tribes. Ongoing studies point increasingly to the fact that the mixed metal wastes from these mines continue to have multigenerational impacts to the health of Native tribes. Our work and others have shown that metals can inhibit DNA-repair processes [96, 97••, 98, 99, 100] and induce immunotoxicity [101–104], providing important clues into the mechanisms by which these mixtures affect health. Through understanding the mechanisms by which these effects occur and understanding the role of exposures in the health disparities seen in these communities, effective interventions can be designed to protect populations for the decades likely to remain before the mine waste is removed or remediated.
